# Effects of age and sex on the distribution and symmetry of lumbar spinal and neural foraminal stenosis: a natural language processing analysis of 43,255 lumbar MRI reports

**DOI:** 10.1007/s00234-021-02670-6

**Published:** 2021-02-16

**Authors:** Michael Travis Caton, Walter F. Wiggins, Stuart R. Pomerantz, Katherine P. Andriole

**Affiliations:** 1 Brigham and Women’s Hospital and Harvard Medical School, Boston, MA USA; 2 Present Address: University of California San Francisco, 505 Parnassus Avenue, L352, CA 94117 San Francisco, USA; 3 Present Address: Duke University, Durham, NC USA; 4 Massachusetts General Hospital and Harvard Medical School, Boston, MA USA; 5 MGH and BWH Center for Clinical Data Science, Boston, MA USA

**Keywords:** Spinal stenosis, MRI, Neuroradiology, Lumbar spine, Natural language processing, Neural foramen, Degenerative disease

## Abstract

**Purpose:**

The purpose of this study is to investigate relationship of patient age and sex to patterns of degenerative spinal stenosis on lumbar MRI (LMRI), rated as moderate or greater by a spine radiologist, using natural language processing (NLP) tools.

**Methods:**

In this retrospective, IRB-approved study, LMRI reports acquired from 2007 to 2017 at a single institution were parsed with a rules-based natural language processing (NLP) algorithm for free-text descriptors of spinal canal stenosis (SCS) and neural foraminal stenosis (NFS) at each of six spinal levels (T12-S1) and categorized according to a 6-point grading scale. Demographic differences in the anatomic distribution of moderate (grade 3) or greater SCS and NFS were calculated by sex, and age and within-group differences for NFS symmetry (left vs. right) were calculated as odds ratios.

**Results:**

Forty-three thousand two hundred fifty-five LMRI reports (34,947 unique patients, mean age = 54.7; sex = 54.9% women) interpreted by 152 radiologists were studied. Prevalence of significant SCS and NFS increased caudally from T12-L1 to L4-5 though less at L5-S1. NFS was asymmetrically more prevalent on the left at L2-L3 and L5-S1 (*p* < 0.001). SCS and NFS were more prevalent in men and SCS increased with age at all levels, but the effect size of age was largest at T12-L3. Younger patients (< 50 years) had relatively higher NFS prevalence at L5-S1.

**Conclusion:**

NLP can identify patterns of lumbar spine degeneration through analysis of a large corpus of radiologist interpretations. Demographic differences in stenosis prevalence shed light on the natural history and pathogenesis of LSDD.

## Introduction

Lumbar spine degenerative disease (LSDD) resulting in spinal canal stenosis and neural foraminal stenosis (SCS, NFS) is a major cause of disability and drives a significant portion of healthcare costs [1]. Knowledge of the epidemiology of LSDD is thus important not only for clinical decision-making on the patient level but also healthcare policy and delivery at a systems level. Gold standard normative measurements of SCS and NFS from cadaveric studies have limited generalizability as they do not directly correlate with the selected population undergoing MRI evaluation. Additionally, how these measurements correlate with in vivo imaging techniques is uncertain [2]. Data from studies of general population samples including the Framingham and Wakayama cohorts provide an estimate of the distribution and relative severity of LSDD but with limited statistical power to detect small but clinically important differences [3, 4].

Lumbar MRI (LMRI) is the gold standard imaging tool for LSDD and is a key criterion for treatment planning. Although there are conflicting data on the prospective correlative value of LMRI with clinical symptoms [5, 6], there is evidence of reasonable inter-reader agreement for MRI; thus, it is a suitable modality for investigating LSDD patterns in heterogeneous populations [7]. Best-practice reporting standards for LMRI have been described with multidisciplinary consensus, favoring a systematic, level-by-level approach, using consistent and accurate terminology [8–10]. These features allow a rich textual description of LSDD to be extracted from the radiology report text. The relative structure of LMRI reporting, reflecting from the level-by-level nature of disease, is also relatively more amenable to natural language processing (NLP) analysis compared to that in other organ systems, potentiating analysis of very large datasets.

The objective of this study is to report the anatomic and demographic distribution of potentially actionable lumbar stenosis—defined as “at least moderate stenosis” in a symptomatic patient cohort by analyzing radiologic stenosis grades from a 10-year institutional archive of LMRI reports. By investigating such a large dataset, we hope to overcome limitations of previous prevalence studies. Because of the near ubiquity of at least mild LSDD in the higher age groups, distinguishing “potentially actionable” disease is a key manner in which LMRI adds value to clinical decision-making for surgeons [11]. Although the correlation of imaging findings and clinical features is imperfect, there is reasonable clinical-radiographic concordance for stenosis rated as “moderate” or greater [6, 12]. We therefore sought to focus on this benchmark of stenosis severity which may prompt surgical referral and intervention.

## Materials and methods

### Study design

This study was a retrospective, observational study performed with Institutional Review Board approval and in compliance with the Health Insurance Portability and Accountability Act (HIPAA). Informed consent was waived.

We queried the reporting archives for LMRI examinations from 2007 to 2017 performed at a single large, urban, academic medical center and its affiliate satellite imaging facilities, yielding 43,255 studies. Studies were not screened for chief complaint/indication from the referring provider or medical history and comorbidity.

### Natural language processing extraction of radiologist stenosis grading

The full text of each radiology report was passed through a customized, rules-based natural language processing (NLP) algorithm for automatic extraction of stenosis grading on a per-level basis, building on principles described by Tan et al. [13]. Using regular expressions (RegEx), report text was parsed into separate principle report sections (Indication, Technique, Findings, Impression). The “Findings” section was further parsed into texts blocks for each discrete level from T12-L1 through L5-S1. The NLP algorithm was built using an empirically developed dictionary of syntactic and semantic rules and common radiology terminology specific to lumbar spine imaging. This system enabled the algorithm to recognize a wide spectrum interchangeable words (e.g., “neural foramen”, “neural foramina”, “neuroforaminal”) and tautological phrases (e.g., “mild bilateral neuroforaminal stenosis” and “left and right neuroforaminal stenosis” recognized as equivalent scores). From each level, free-text severity descriptors of central spinal stenosis (SCS), left neuroforaminal stenosis (NFS), and right NFS were extracted and mapped to a 6-point severity grading scale (0 = “Normal”, 1 = “Mild”, 2 = “Mild to Moderate”, 3 = “Moderate”, 4 = “Moderate to Severe”, 5 = “Severe”). An iteratively assembled dictionary of non-standard terms (e.g., “marked” or “minimally”) facilitated mapping of non-standard terms to the 6-point grading scale. Population of values thus resulted in a 6 × 3 matrix comprising a total of 18 “level instances” (e.g., L1-L2 right NFS, L1-2 left NFS, etc.). For failure cases, in which the algorithm could not identify a reported stenosis severity grading, we applied a score of “0” (normal), under the premise that normal anatomy may be presumed by the absence of specific comment by the radiologist. To test the accuracy of the model, we randomly selected 100 studies out of the full dataset (*n* = 43,255) and manually reviewed the radiology reporting text to assess for discrepancy or error. For each case, the report text was manually reviewed and assigned a severity score (“normal” to “severe”, 0–5) by a radiologist. Scores were considered concordant if NLP and manual review matched exactly and any degree of discordance was considered unsuccessful.

### Statistical analysis

Comparisons between demographic groups (age, sex) and comparisons within each group by laterality (left vs. fight) for differences in potentially actionable LSDD—defined as “moderate (grade 3) or greater”—prevalence were calculated as an odds ratio (OR) with 95% confidence intervals and statistical differences determined by the chi square test. All statistical analysis was performed using R v3.6.2 (The R Foundation for Statistical Computing). Odds ratios were also calculated to test for symmetry in distribution of NFS (left and right) and are reported with the null assumption (OR = 1) that NFS distribution is equal bilaterally. *p* values of less than 0.05 were considered significant.

## Results

### Lumbar MRI exam characteristics

In total, 43,255 LMRI exams performed between 2007 and 2017 for 34,947 unique patients were included in the analysis. Exams were performed on 30 different scanners across 23 imaging sites within the Massachusetts General Hospital network by 162 attending radiologists and 354 unique trainees including residents and fellows. Nineteen thousand four hundred ninety-five (45.1%) were men and 23,758 (54.9%) were women. The mean age was 54.7 (interquartile range = 26–80 years). The exams were divided into three age categories: less than 50 (*n* = 16,267), 50 to 70 (18,077), and greater than 70 (*n* = 8911).

### NLP algorithm validation

The NLP accuracy was 94.8% in a random sample of 100 LMRI reports (93 misclassifications out of 1800 level instances). At individual levels, NLP accuracy ranged from 86.0% at right L5-S1 to 100% in 5/18 level instances (27.8%).

### Distribution and severity of LSDD in the study cohort

**Fig. 1:**
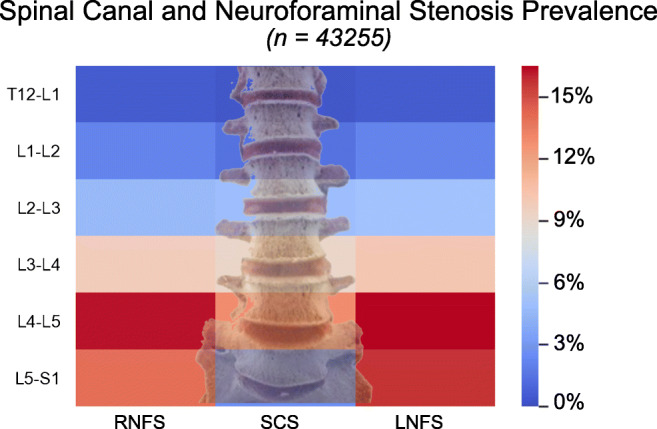
Prevalence of moderate or greater spinal stenosis in the full cohort of LMRI exams (*n* = 43,255), organized by level. RFS, right-sided neuroforaminal stenosis; SCS, central spinal stenosis; LFS, left-sided neuroforaminal stenosis. The prevalence of SCS and LFS was highest at L4-5. Color-coding represents absolute frequencies for each level instance

Distribution of potentially actionable SCS and NFS—defined as “at least moderate”—in the full 10-year cohort (*n* = 43,255 distinct LMRI studies) is shown graphically in Fig. 1. We observed the highest rates of moderate or greater SCS at L4-5 (12.9%) followed by L3-4 (9.1%), L2-3 (4.9%), L5-S1 (2.6%), and L1-2 (1.4%) with the lowest prevalence at T12-L1 (0.4%). For this same overall cohort, the highest prevalence of neural foraminal stenosis (*n* = 86,510 individual foramina) was L4-5 (16.4%) followed by L5-S1(14.8%), L3-4 (9.9%), L2-3 (4.9%), L2-1 (2.2%), and T12-L1 (0.6%).

**Fig. 2:**
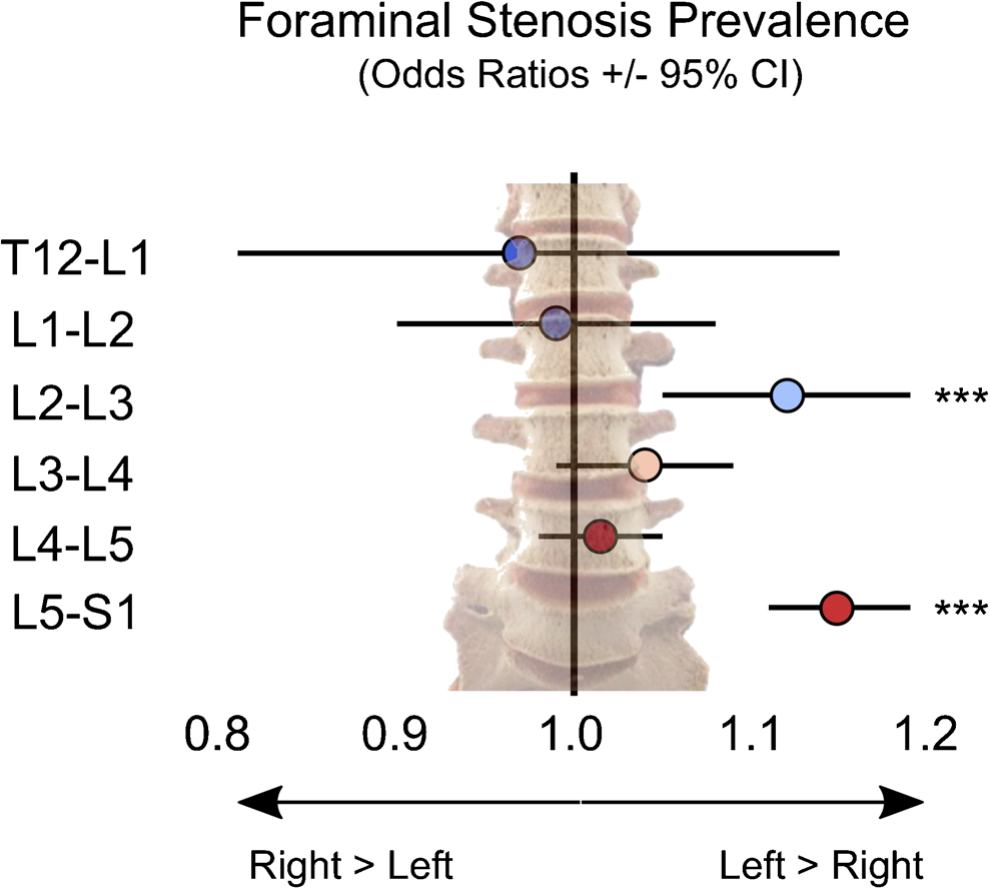
Asymmetric distribution of moderate or greater neural foraminal (NF) stenosis in the lumbar spine. The odds ratio of disproportionate NF stenosis prevalence is plotted for each level (OR 1.0 = equal probability left and right). The horizontal bars indicate the standard error. The OR favored left greater than right NF stenosis prevalence for L2-3 (OR = 1.12, 95% CI (1.05, 1.19), *p* < 0.001) and L5-S1 (OR = 1.15, 95% CI (1.11, 1.19), *p* < 0.001). The relative prevalence of neural foraminal stenosis is color-coded (red, higher prevalence; blue, lower prevalence)

In this large cohort (*n* = 43,255), there is asymmetry in the prevalence of NFS by laterality, shown schematically in Fig. 2. We found a statistically significantly higher prevalence of NFS at the L2-3 (*p* < 0.001) and L5-S1 (*p* < 0.001) levels on the left side. Left-sided stenosis was also disproportionately prevalent at L3-4 though not statistically significant (*p* = 0.09). No statistical difference between left and right was seen for the remaining levels.

### Sex differences in LSDD

**Fig. 3:**
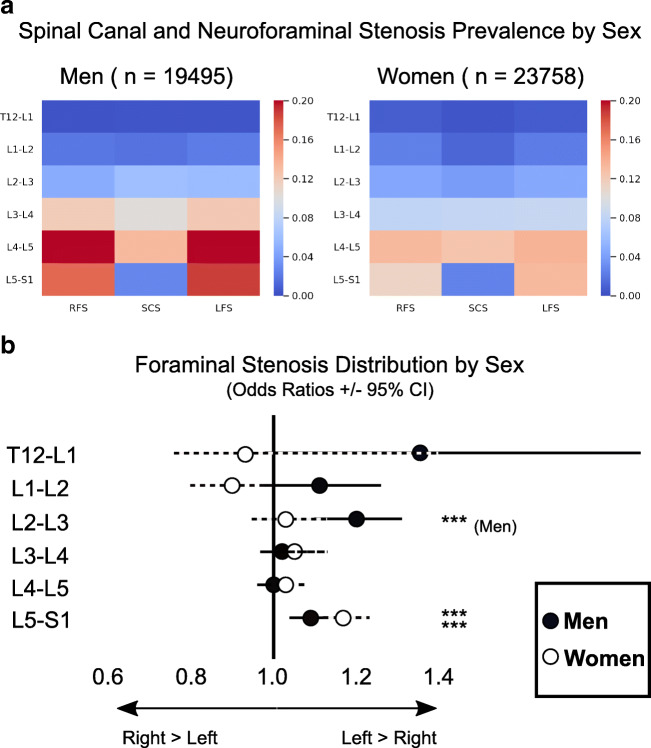
Prevalence of potentially actionable LSDD by sex. Central spinal stenosis (SCS) and neural foraminal stenosis (NFS) were more prevalent in men at all levels (**a**). Color indicates the absolute prevalence of moderate or greater SCS and NFS at each level. The pattern of distribution of neural foraminal stenosis in men and women shown as odds ratios with 95% confidence interval indicated by the solid line (men) and dashed line (women) (**b**). *** indicates *p* < .001

The relative prevalence of significant SCS and NFS by sex is shown in heat maps in Fig. 3a. SCS prevalence ranged from 0.4 to 12.6% (T12-L1, L4-5) in women and from 0.4 to 13.3% (T12-L1, L4-5) in men. The prevalence of potentially actionable SCS was higher for men than women at L1-2, L2-3, L3-4 (*p* < 0.001), and L4-5 (*p* 0.03). The only level which showed higher SCS prevalence for women rather than men was T12-L1, but this difference was not statistically significant (*p* = 0.38).

Sex differences in NFS prevalence are also shown as heat maps in Fig. 3a and laterality distribution of NFS are shown in Fig. 3b (OR +/− 95% CI). NFS stenosis prevalence ranged from 0.8 to 13.3% (T12-L1, L4-5) in women and from 0.3 to 19.9% (T12-L1, L4-5) in men. NFS was more prevalent in women than men at T12-L1 for both sides (right NFS OR 2.57, left NFS OR 1.72, *p* < 0.001 for both) and for L1-L2 on the right side only (right NFS OR 1.26, *p* < 0.001). Right and left NFS were more prevalent in men at the remaining levels except L2-3 which showed no statistical between-group difference (*p* = 0.08 for right NFS, *p* = 0.74 left NFS). The greatest magnitude of difference between men and women was at T12-L1, with higher prevalence of NFS in women relative to men (LFS OR 1.72, RFS OR 2.57, *p* < .001 for both).

We then queried the symmetry of NFS distribution for both men and women (Fig. 3B). Laterality differences presented as OR (+/− 95% CI). For men, there was disproportionate NFS prevalence on the left at L2-3 (OR 1.2, 95% CI (1.1–1.31), *p* < 0.001) and L5-S1 (OR 1.09, CI 1.04–1.13, *p* < 0.001). There was a trend toward greater left NFS at T12-L1 (*p* = .06). Right NFS was not more prevalent at any level in men. For women, the only statistically significant asymmetry in NFS was left-predominance at L5-S1 (OR 1.17, CI 1.12–1.23 *p* < 0.001). There was a trend toward higher left NFS at L3-4 (*p* = 0.08) and higher right NFS at L1-2 (*p* = 0.08).

### Age differences in LSDD

**Fig. 4:**
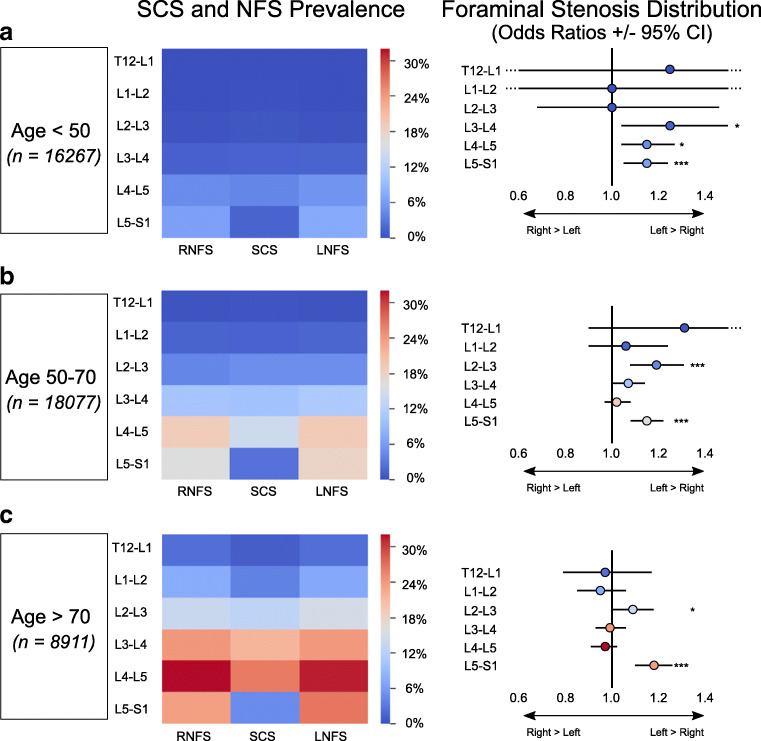
Prevalence of moderate or greater LSDD by age group (**a** < 50, **b** 50–70, and **c** > 70). The highest relative proportion of NFS was at L5-S1 for age < 50 and at L4-5 for both age 50–70 and age > 70 groups. Asymmetry in neural foraminal stenosis by age group, measured as odds ratios (OR). OR > 1 indicates left greater than right, and OR < 1 indicates right greater than left. Statistical significance indicated by *p < .05, ***p < .001. The color of each level corresponds to the overall age-group frequency at a given level (absolute prevalence in the sub-population)

**Fig. 5:**
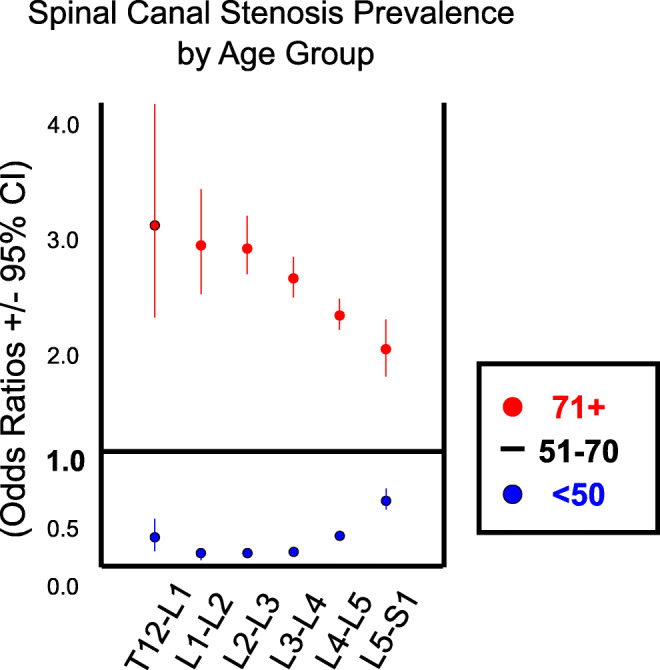
Differences in moderate or greater spinal canal stenosis (SCS) prevalence by age group shown as odds ratios, normalized to the middle-age patient population (OR = 1). The relative distance from the horizontal black line indicates the effect size of age as the change in relative frequency between age groups; the smallest change from < 50 to 51–70 and from 51–70 to 71+ is at L5-S1. Larger shifts in disease prevalence are seen for L1-2, L2-3, and L3-4 among the age groups

The relative prevalence of SCS and NFS was analyzed across three age groups (< 50, 50–70, > 70), and the absolute prevalence of potentially actionable disease across these groups is shown in Fig. 4a, b, and c. Absolute values of SCS prevalence were lowest for all levels in the youngest patients (< 50 group) with prevalence ranging from 0.1 to 4.3% (T12-L1, L4-5). Figure 5 shows differences in SCS prevalence between age groups as odds ratios, allowing estimation of the effect size of the difference. SCS prevalence for the intermediate age group (50–70) ranged from 0.4 to 14.1% (T12-L1, L4-L5) and was statistically higher than the < 50 group (*p* < 0.001 all levels). The largest magnitude of increase was seen at L4-5 (+ 9.8%). In the older age group (age > 70), SCS prevalence ranged from 1.1 to 26.3% (T12-L1, L4-L5). SCS prevalence was higher in the > 70 group than the 50–70 group for each level (*p* < 0.001, all cases). The largest magnitude of increase from 50–70 to 70+ was at L4-5 (+ 12.2%). The effect size of age group is shown visually in Fig. 5 with OR 1.0 corresponding to the intermediate group. This revealed larger effect size age at higher levels (T12-L1, L1-L2, L2-L3, and L3-L4) despite lower absolute prevalence at these levels.

Intra-group differences in significant NFS by age are shown in Fig. 4. For the < 50 age group, the lowest prevalence of NFS was at T12-L1 (0.03% LNFS, 0.02% RNFS), and the highest prevalence was at L5-S1 (7.3% LNFS, 6.4% RNFS). For the intermediate age group (50–70 years old), lowest rates of NFS were at T12-L1 (0.4% LNFS, 0.3% RNFS). In contrast to the < 50 group, the highest prevalence of NFS for those aged 50–70 was at L4-5 (19.3% LNFS, 19.0% RNFS) rather than L5-S1. NFS prevalence was higher at all levels in the 50–70 group relative to the < 50 group (*p* < 0.001, all cases). For the > 70 group, NFS prevalence ranged from 2.3 to 32.0% (T12-L1, L4-L5). NFS was higher in the > 70 group than the 50–70 group at all levels (*p* < 0.001, all cases).

Symmetry of NFS distribution, presented as OR +/− CI, are shown in Fig. 4, and individual level instance data are compared to the right side (OR 1.15, *p* < 0.001) with borderline statistical significance at L3-4 (OR 1.25, *p* = 0.02) and L4-5 (OR 1.15, *p* = 0.01). In the 50–70 group, LNFS was more prevalent than RNFS at L2-3 (OR 1.19, *p* < 0.001) and L5-S1 (OR 1.15, *p* < 0.001) with borderline left asymmetric prevalence at L3-4 (OR 1.07, *p* = 0.06). In the > 70 age group, LNFS was higher than RNFS at L5-S1 only (OR1.18, *p* < 0.001) with trend toward preferential LFNS distribution at L2-3 (OR 1.09, *p* = 0.05).

## Discussion

This study describes an NLP-driven approach toward understanding the interaction between demographics and distribution of LSDD through large-scale analysis of radiology reports for LMRI examinations. Our results are consistent with findings of previous general population samples (Framingham, Wakayama, and others) showing the overall highest prevalence of SCS at the L4-5 level [4, 14]. We also identify sex differences in prevalence which have been reported in a highly cited community-based study of fewer than 200 patients [15]. Our method not only reproduces these important findings on a larger scale but provides an estimate of the effect size of age and sex on LSDD prevalence on a level-by-level basis (Figs. 3, 4, and 5) [4, 15]. Moreover, our study builds on previous work by evaluating a larger cohort, different mechanism of documentation, and inclusion of a broader age range. In addition, we also estimate the prevalence of NFS revealing left-right asymmetry at L3-4 and L5-1, which has not previously been reported in the radiology or surgical literature.

We also observed an interesting change in SCS when comparing age groups: Younger patients (< 50) showed relatively higher prevalence of NFS at L5-S1, whereas older age groups showed highest prevalence of LSDD for SCS at L4-5. The relative effect size of increasing age (shown as odds ratios, the distance between red and blue points in Fig. 5) is higher for the upper lumbar levels and lower at L5-S1, suggesting that L5-S1 disease may be pathophysiologically distinct from age-related effects seen at other levels. One theory is that L5-S1 NFS is more prevalent due to sampling bias of younger patients with premature NFS due to L5-S1 pars defects (spondylolysis), which typically develop in adolescence and are associated with load-bearing exercise and sports activity. Spondylolysis at L5-S1 is thought to arise from repetitive microtrauma and can result in anterolisthesis which can narrow the sagittal diameter of the neural foramen [16]. We speculate that the rising incidence of degenerative, non-spondylolytic disease at higher age groups in effect “washes out” the NFS related to pars defects in this study; however, we did not evaluate for spondylolysis in the present analysis.

The mechanism underlying the finding of asymmetric distribution of foraminal stenosis is not clear. This observation is consistent with an anatomic-pathology study which found significantly thicker L4-5 and L5-S1 ligamentum flavum [17]. One potential etiology is so-called ‘transitional’ lumbosacral anatomy (TLSA), which is highly prevalent (up to 30–35% of population) but often asymptomatic [18]. TLSA is typically unilateral and predisposes patients to adjacent segment LSDD (L4-5), and a recent study of incidental TLSA suggests correlation with LSDD at higher lumber levels (L2-3, L3-4) [19]. The left-sidedness of the aorta is another interesting potential mechanism, albeit more speculative in nature. Age and atherosclerosis influence the pressure wave transmitted by pulsations of the aorta which would asymmetrically affect the spinal column [20]. An association between aortic calcification and LSDD at the adjacent spinal level has also been observed, suggesting a mechanical coupling of these structures which could predispose to asymmetric degeneration [21]. Interestingly, the level of aortic bifurcation differs in individuals with TLSA, raising the possibility of an interaction between TLSA, the aorta, and LSDD asymmetry [22]. An alternate hypothesis suggests that atherosclerotic disease of aortic branches results in chronic ischemic injury of the disc and endplates, thereby accelerating the degenerative process [23].

Chronic remodeling of the foramina due to coronal imbalance in the setting of scoliosis is another plausible mechanism of LSDD asymmetry [24]. The distribution of mid-thoracic scoliosis in adolescents is not evenly distributed as shown in a study of over 8000 school children [25]. Another potential mechanism is asymmetry in handedness in the population. Right-hand dominance is more common globally and known to contribute to asymmetric occupational injury in the upper extremities [26]. In elite tennis players, for example, imbalanced use of paraspinal muscles and axial skeletal extension due to handedness has been associated with lower back pain [27]. This form of lifelong ergometric imbalance in occupation and activities has been posited as a source of chronic degenerative musculoskeletal injury by Kumar due to differential fatigue or cumulative mechanical loading [28]. This could in turn explain the foraminal asymmetry seen in our study; however, we did not evaluate for the presence or directionality of scoliosis in the present analysis. Alternatively, handedness/occupational factors contributing to imbalanced LSDD may exert a selection bias on patients presenting for LMRI.

Our method of NLP-driven analysis has several strengths, the chief one being the large sample size. First, by including a large corpus with the interpretation of 152 different attending radiologists over a 10-year period could mitigate the impact of inter- and intra-reader variation [29] by leveraging the statistical “Law of Large Numbers” to estimate the mean across our study population. It is important to note that we did not analyze reports from CT or CT myelographic imaging of the lumbar spine in our analysis, which may result in some degree of sampling bias, as patients who were unable to undergo MRI—potentially due to claustrophobia or implanted devices—were not included. Despite this potential sampling bias, we believe our sample is representative of the referral base for LMRI in our institution. Moreover, the large sample size powers this study to detect small differences, even when stratified by sub-populations.

The use of radiology reports to estimate disease epidemiology is limited by several factors. The NLP algorithm empowers a much larger corpus to be analyzed, but its classification is imperfect. Our institution implemented non-mandatory semi-structured reporting before the study period; however, a subset of radiologists did not utilize the semi-structured reporting templates. Therefore, our assumption that “non-mention” of SCS/NFS at a given level may underestimate true disease prevalence. Furthermore, our rules-based algorithm relied on a manually assembled dictionary mapping non-standard terminology for stenosis grading to our 6-point standard scale. This methodology is imperfect and may affect our estimate of the severity distribution of LSDD. Although our algorithm documents 6 grades of stenosis, we lumped those greater than 3 to de-noise the data and focus our analysis on the prevalence of potentially actionable LSDD.

Another limitation of our study is that the study cohort is not a true sampling of the general population as only patients with symptoms substantial enough to warrant imaging are studied. In the USA, for example, refractory back pain or neurologic symptoms must persist despite best conservative therapy for at least 6 weeks for reimbursement by the Centers for Medicare and Medicaid Services for LMRI [30, 31]. The generalization of these findings to asymptomatic patients is therefore limited.

In summary, we report a contemporary estimate of the prevalence of potentially actionable lumbar stenosis in a 10-year series of over 43,000 MRI studies. This work provides an estimate of the prevalence of lumbar spinal stenosis in a symptomatic cohort and reveals asymmetry in neural foraminal LSDD which may be due to occupational or biomechanical factors accrued over the lifespan. Future work may lead to establishment of a normative database for LSDD severity by age and sex, potentially facilitating improved disease management.

## Abbreviations

LMRI: Lumbar spine MRI
SCS: Spinal canal stenosis
NFS: Neural foraminal stenosis
LSDD: Lumbar spine degenerative disease
NLP: Natural language processing
TLSA: Transitional lumbosacral anatomy ()

## Declarations

The author(s) declare that they had full access to all of the data in this study and the authors take complete responsibility for the integrity of the data and the accuracy of the data analysis.
